# Effects of β-glucans on fatigue: a systematic review and meta-analysis

**DOI:** 10.1038/s41430-025-01567-4

**Published:** 2025-01-28

**Authors:** Masahiro Muroya, Kumiko Nakada, Kazushi Maruo, Koichi Hashimoto

**Affiliations:** 1 Department of Clinical and Translational Research Methodology, Institute of Medicine, University of Tsukuba, Ibaraki, Japan; 2 Department of Biostatistics, Institute of Medicine, University of Tsukuba, Ibaraki, Japan

**Keywords:** Randomized controlled trials, Nutrition

## Abstract

Several clinical trials suggest that β-glucans may reduce feelings of fatigue, however the results of clinical trials are inconsistent. Additionally, no systematic reviews or meta-analyses have assessed the effects of β-glucans on fatigue. Therefore, this study investigates the effects of β-glucans on fatigue in healthy subjects through a systematic review and meta-analysis. PubMed, Web of Science, and the Cochrane Central Register of Controlled Trials (CENTRAL) were searched from database inception to March 15, 2024. The inclusion criterion was a randomized controlled trial (RCT) investigating the effects of β-glucans on healthy subjects’ fatigue, vigor, and mood state. To assess risk of bias, we employed the Cochrane risk of bias tool. A random-effects meta-analysis was conducted for the standardized mean difference (SMD). Sixteen RCTs with a total of 1,449 participants were included, and 12 studies provided data suitable for meta-analysis. Meta-analysis revealed that β-glucans significantly reduced feelings of fatigue (SMD = −0.32, 95% CI = −0.53 to −0.12; *p* = 0.0021), increased vigor (SMD = 0.46, 95% CI = 0.26–0.66; *p* < 0.0001), and improved mood state (SMD = 0.32, 95% CI = 0.11–0.53; *p* = 0.0026) compared to the placebo group. The results of the systematic review and meta-analysis indicate that β-glucans may be effective in reducing feelings of fatigue in healthy individuals. However, the number of studies included is insufficient, suggesting that further clinical trials are needed to validate this effect.

## Introduction

Fatigue is a common physiological phenomenon encompassing both physical and mental components, experienced by many individuals on a daily basis. Symptoms of fatigue include tiredness, weakness, and a lack of energy [1]. In healthy individuals, causes of fatigue are often linked to occupation, social events, and personal lifestyle [2], with significant economic implications for society [3]. Given this context, there is a growing need to develop food products scientifically proven to alleviate feelings of fatigue.

β-glucans are dietary fibers found in various sources such as mushrooms, barley, and yeast. They consist of polysaccharides composed of D-glucose through β-glycosidic linkages. The molecular structure of β-glucans varies depending on their source, with bacterial β-glucans containing β-1,3 linkages, cereal β-glucans containing β-1,3-1,4 linkages, and those from seaweeds and yeasts containing β-1,3-1,6 linkages [4]. β-glucans are categorized into grain-derived and non-grain-derived types based on their source. Grain-derived β-glucans have been associated with cholesterol and blood sugar level reduction, whereas non-grain-derived β-glucans are known for their immunomodulatory effects [5]. Receptors for β-glucans, such as Dectin-1 and complement receptor 3 (CR3), have been identified [6]. It has been reported that binding of β-glucans to Dectin-1 activates immune cells, thereby inducing innate and acquired immunity [7].

Several clinical trials have investigated the effects of β-glucans on fatigue. In a study involving marathon runners, the administration of 500 mg of yeast-derived β-glucan per day for four weeks significantly reduced feelings of fatigue compared to the placebo group [8]. Similarly, in healthy subjects experiencing fatigue in daily life, the consumption of 350 mg of euglena-derived β-glucan per day for four weeks led to a significant reduction in both physical and mental fatigue compared to the placebo group, although no significant difference was observed in overall fatigue [9]. Conversely, in healthy subjects with LDL-cholesterol levels ranging from 3 to 5 mmol/L, the administration of 3000 mg of oat-derived β-glucan per day for four weeks did not result in a significant difference in the prevalence and severity of fatigue compared to the placebo group [10]. Thus, while various clinical trials have been conducted on the effects of β-glucans on fatigue, the results have shown inconsistency.

Despite numerous systematic reviews focusing on β-glucans, none have specifically addressed their effects on fatigue. Previous studies have explored β-glucans’ role as a biomarker in diagnosis [11], their potential as therapeutic adjuvants in cancer patients [12], their impact on blood glucose and lipid levels [13], and their effects on upper respiratory tract infections [14]. However, the impact of β-glucans on fatigue remains unclear, as no systematic review has specifically examined this aspect. Understanding the effects and mechanisms of β-glucans on fatigue could offer novel strategies for alleviating fatigue symptoms. Therefore, this study aimed to investigate the effects of β-glucans on fatigue in healthy subjects through a systematic review and meta-analysis.

## Materials and methods

This study adhered to the Preferred Reporting Items for Systematic Reviews and Meta-Analyses (PRISMA) guidelines [15].

### Search strategies

PubMed, Web of Science, and the Cochrane Central Register of Controlled Trials (CENTRAL) were systematically searched from their inception to March 15, 2024, with no language restrictions applied. The search terms were comprised of three groups: (1) β-glucan, (2) Randomized Controlled Trial, and (3) fatigue. The detailed search strategy for this systematic review and meta-analysis is provided in the Supplementary Material (Supplementary Table S1). Additionally, hand searches were conducted for comprehensive coverage.

### Study selection

Two researchers (MM and KN) independently conducted the study selection process. Any discrepancies were resolved through discussion with a third researcher (KH). The inclusion criteria were based on the PICOS (patients, intervention, comparison, outcomes, and study design) framework. The population included healthy subjects, with no restrictions on age, gender, or ethnicity, while individuals suffering from disease were excluded. The intervention involved the oral intake of foods and supplements containing β-glucans, with no limitations on the source, amount, or duration of β-glucan intake. The comparison control group was not restricted. Outcomes comprised studies assessing the effects of β-glucans on fatigue, vigor, and mood state. The study design was restricted to randomized controlled trials. Animal studies, review articles, and studies where β-glucans were not the sole dietary intake were excluded.

### Data extraction

The following data were extracted from the included articles: author, country, year of publication, target population characteristics (including age), intervention and control details, number of participants, β-glucan characteristics (source, intake, duration), and outcome scale and results (specifically fatigue, vigor, and mood state). To ensure accuracy and prevent misinterpretation, special attention was paid to the direction of the effect of the scales used in each study. The primary outcomes analyzed were fatigue, vigor, and mood state scores following β-glucan and placebo interventions. In cases where results were presented graphically, they were quantified using WebPlotDigitizer [16]. Data extraction and conversion adhered to the recommendations outlined in the Cochrane Handbook [17]. For data presented as standard errors (SE), the standard deviation (SD) was calculated by multiplying the SE by the square root of the number of samples. For data presented with 95% confidence intervals, SD was obtained by dividing the width of the confidence interval by 3.92 and multiplying by the square root of the sample size. For data reporting the difference in means (MD) between groups with a given *p*-value, the t statistic was obtained from the t distribution table and converted to SD by dividing MD by the t statistic; the same SD was applied to both groups. If SD was not provided or calculable, SD from studies utilizing the same scale was utilized, with preference given to the mean if multiple studies used the same scale.

### Quality assessment

Risk of bias in randomized trials was assessed using the Cochrane risk of bias tool for randomized trials (RoB2) [18]. This assessment covered five domains: bias risk arising from randomization processes, deviations from intended interventions, missing outcome data, outcome measurement, and selection of reported results. For crossover trials, bias risk from period and carryover effects was also evaluated. Overall bias risk was categorized as follows: (1) Low risk of bias: No bias concerns across all domains. (2) Some concerns: At least one area with potential bias concerns. (3) High risk of bias: At least one domain presents a high risk of bias.

The Grading of Recommendations Assessment, Development and Evaluation (GRADE) approach was employed to evaluate the certainty of evidence for each outcome [19, 20]. Since the study design is a randomized controlled trial, the certainty of evidence starts as high and is then evaluated based on five domains that may lower the grade: risk of bias, inconsistency, indirectness, imprecision, and publication bias. Certainty of evidence was graded as high, moderate, low, or very low.

### Data synthesis and statistical analysis

Data analysis was conducted using the meta package [21] of R version 4.2.1 (R Core Team, Vienna). A random-effects meta-analysis was performed to estimate the standardized mean differences (SMD) between the β-glucan intervention and placebo groups. When multiple post-intervention data points were available, outcomes reported at the end of the intervention period were utilized. In cases where there were different β-glucan intake groups within a study, data from the group with the highest intake were included in the analysis. For crossover studies, due to insufficient information, data from both intervention and control periods were combined and treated as if they were from parallel studies. We conducted a subgroup analysis based on the outcome evaluation methods. Heterogeneity across studies was assessed using the I^2^ statistic. Publication bias was evaluated using funnel plots, Egger’s test, and Begg’s test. The significance level for statistical tests was set at 0.05, and a confidence level of 95% was applied to confidence intervals (CI).

## Results

### Study selection

**Fig. 1:**
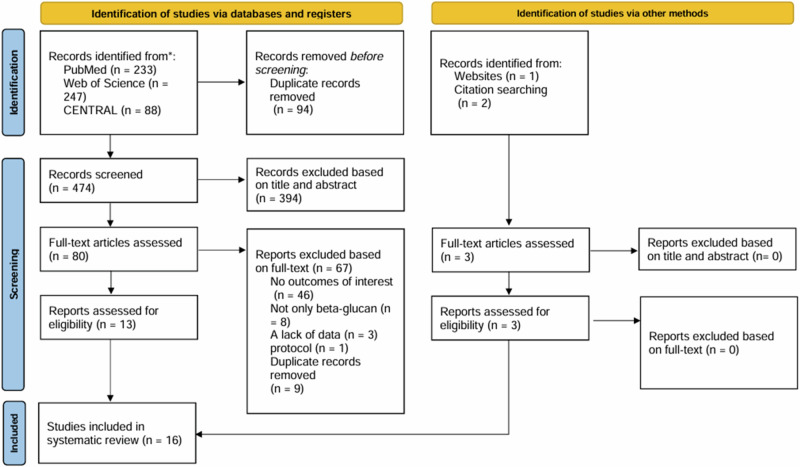
PRISMA flow diagram of study selection. PRISMA flow diagram summarizing the results of the search and selection processes.

Following the PRISMA guidelines [15], the process of study selection is depicted in Fig. 1. The literature search identified a total of 571 studies, comprising 233 articles from PubMed, 247 from Web of Science, 88 from CENTRAL, and 3 from Handsearching. After removing duplicates, 477 unique studies remained. Subsequently, 394 studies were excluded after screening based on title and abstract, resulting in 83 studies for further evaluation. Upon full-text screening, 67 studies were excluded, ultimately leaving 16 studies for inclusion in the systematic review. Among them, 12 studies were eligible for the meta-analysis.

### Study characteristics

Table 1 presents the characteristics of the included studies. These studies were conducted across various countries, including Canada, Japan, the USA, Norway, and Germany, spanning publication years from 2009 to 2022, with a total coverage of 1449 individuals. Although the target population across studies was generally healthy, specific characteristics varied. For instance, one study focused on individuals with qi deficiency [22], another on those with LDL-cholesterol levels between 3 and 5 mmol/L [10], and additional studies targeted individuals with decreased vitality, sleep quality [23], feelings of fatigue [9], engagement in aerobic or endurance exercise [24, 25], recurrent upper respiratory tract infections [26], marathon runners [8, 27], allergies such as ragweed or hay fever [28], and varying levels of psychological stress [29, 30]. Furthermore, one study focused on individuals with a history of colds [31]. In all cases, the comparison group received a placebo. Study designs included 13 randomized, double-blinded, placebo-controlled parallel studies, and three randomized, double-blinded, placebo-controlled crossover studies. The sources of β-glucans varied, including oats, yeast, euglena, shiitake mushroom, and combinations thereof. Daily intake ranged from 1 mg to 3000 mg, with durations varying from 10 days to 16 weeks. Fatigue, vitality, and mood state were the primary outcomes assessed, utilizing various measurement tools such as the Patient-Reported Outcomes Measurement Information System (PROMIS), Symptoms Questionnaire, Short-Form Health Survey (SF-12/SF-36), Visual Analogue Scale (VAS), Profile of Mood States (POMS), and Wisconsin Upper Respiratory Symptom Survey (WURSS-24). Additionally, during the intake period, specific tasks or exercises were performed in some studies, including the Uchida-Kraepelin test [32], two-back task, and advanced trail making test (ATMT) [33], as well as treadmill exercises at 55% maximal oxygen uptake in hot and humid conditions [24].

**Table 1:** Characteristics in the included studies.

Study	Country	Participants	Age^a^	Grouping	Dosage^b^	*N* ^c^	Duration	Outcome	Design
Wu [22]	Canada	Healthy subjects with severity score of protective qi deficiency >1.8	18 ~ 65	Yeast, Reishi, Shiitake (Proglucamune®)	~200 mg	70	12 weeks	PROMIS (mental health)	RCT
				Placebo		68			
Wolever [10]	Canada	Healthy subjects with LDL-cholesterol between 3.00 and 5.00 mmol/L	47.6 ± 11.4	Oat β-glucan	3000 mg	96	4 weeks	Symptoms Questionnaire (Fatigue)	RCT
				Placebo		95			
Nakashima [23]	Japan	Healthy subjects with reduced motivation based on the vitality measured by SF-36 and poor quality of sleep based on the PSQI total score	48.0 ± 9.7	(Euglena gracilis powder)	(3000 mg)	20	12 weeks	SF36 (vitality)	RCT
			46.0 ± 10.7	(Euglena gracilis powder)	(1000 mg)	20			
			47.8 ± 11.5	(Euglena gracilis powder)	(500 mg)	19			
			47.3 ± 9.5	Placebo		18			
Kawano [9]	Japan	Healthy subjects with fatigue sensation in daily life	49.2 ± 6.5	Paramylon (Euglena gracilis EOD-1)	350 mg	25	4 weeks	VAS (Overall Fatigue, Physical Fatigue, Mental Fatigue)	RCT
			48.9 ± 10.3	Placebo		27			
Zabriskie [24]	USA	Healthy subjects who reported performing some form of aerobic exercise at least twice per week for the last 12 months	29.9 ± 7.7	Yeast β-glucan (Yestimun®)	250 mg	31	13 days	POMS (Fatigue, Vigor, Total Mood Disturbance)	RCT-Cross over
				Placebo					
Aldwinckle [35]	Norway	Healthy subjects	/	Lentinan (Lentinex®)	1–2 mg	42	4 weeks	VAS (well-being)	RCT
				Placebo		14			
Evans [25]	Canada	Healthy subjects who perform 1.5–3 h/day of endurance exercise or sport, 5–6 days/week	46.8 ± 13.2	Algal β-glucan (BetaVia^TM^ Complete)	367 mg	13	90 days	WURSS-24 (Feeling tired)	RCT
			42.9 ± 12.3	Placebo		14			
Ishibashi [36]	Japan	Healthy men	30 ~ 70	Paramylon (Euglena gracilis EOD-1)	350 mg	7	4 weeks	SF-36v2 (energy/vitality, mental health, MCS)	RCT-Cross over
				Placebo					
Dharsono [26]	Germany	Healthy subjects with at least three URTIs during the last year	37.6 ± 14.3	Yeast β-glucan (Yestimun®)	900 mg	145	16 weeks	SF-12 (psychological sum score)	RCT
			40.5 ± 16.4	Placebo		146			
Ojiri [47]	Japan	Healthy subjects who have a non-strenuous daily lifestyle	22.8 ± 2.2	Yeast β-glucan (Wellmune®)	300 mg	14	10 days	POMS (Fatigue, Vigor)	RCT-Cross over
				Placebo					
McFarlin [27]	USA	Marathon runners	34 ± 9	Soluble yeast β-glucan	250 mg	74	28 days	POMS (Global Mental Health)	RCT
			34 ± 11	Insoluble yeast β-glucan	250 mg	73			
			35 ± 11	Placebo		35			
Talbott [28]	USA	Subjects self-described “moderate” sufferers of ragweed allergy or considered themselves to have “hay fever”	36 ± 11	Yeast β-glucan (Wellmune WGP®)	250 mg	24	4 weeks	POMS (Fatigue, Vigor, Global Mood States),	RCT
			39 ± 9	Placebo		24			
Talbott [29]	USA	Healthy women with moderate levels of psychological stress with a score of 6-10 on the stress survey	41 ± 11	Yeast β-glucan (Wellmune WGP®)	250 mg	39	12 weeks	POMS (Fatigue, Vigor, Global Mood States)	RCT
				Placebo		38			
Talbott [30]	USA	Healthy subjects with moderate levels of psychological stress with a score of 6-10 to high levels of psychological stress with a score of 10 or more in the stress survey	39 ± 11	Yeast β-glucan (Wellmune WGP®)	500 mg	50	4 weeks	POMS (Fatigue, Vigor)	RCT
				Yeast β-glucan (Wellmune WGP®)	250 mg	50			
				Placebo		50			
Talbott [8]	USA	Marathon runners	36 ± 9	Yeast β-glucan (Wellmune WGP®)	500 mg	25	4 weeks	POMS (Fatigue, Vigor, Global Mood State)	RCT
				Yeast β-glucan (Wellmune WGP®)	250 mg	25			
				Placebo		25			
Feldman [31]	USA	Healthy subjects with at least 1 self-reported cold in the last 12 months prior to screening	30 ± 11	Yeast β-glucan (Wellmune WGP®)	500 mg	17	12 weeks	・Diary (Total Tiredness)	RCT
			36 ± 16	Placebo		16		・SF-36v2	

*RCT* a randomized, double-blind, placebo-controlled, parallel study, *PROMIS* Patient-Reported Outcomes Measurement Information System, *SF-12/SF-36* Short-Form Health Survey, *VAS* Visual Analogue Scale, *POMS* Profile of Mood States, *WURSS-24* Wisconsin Upper Respiratory Symptom Survey.

^a^ Age is presented as means ± SD.

^b^Dosage is per day.

^c^*N* is sample size.

### Risk of bias in the included studies

**Fig. 2:**
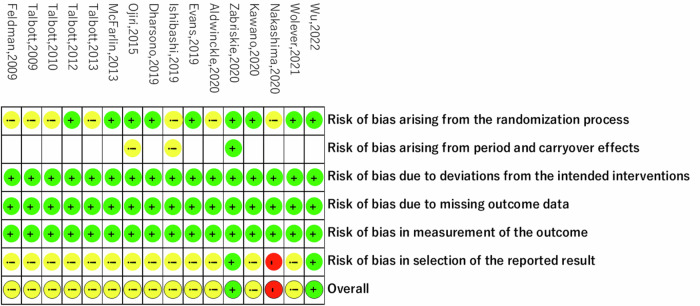
Risk of bias in the included studies. Green cycle: low risk, yellow cycle: some concerns, red cycle: high risk.

Figure 2 illustrates the risk of bias assessed using the Cochrane risk-of-bias tool [18]. Overall, two studies were deemed to have a low risk of bias, while 13 studies were categorized as having some concerns, and one study was considered to have a high risk of bias. Concerning bias related to the randomization process, seven studies raised concerns due to insufficient information. However, all studies demonstrated a low risk of bias regarding deviations from intended interventions, missing outcome data, and measurement of outcomes. Regarding bias in the selection of reported results, 13 studies were noted to have some concerns, with one study assessed as having a high risk of bias, as it reported only partial results for SF-36 and POMS2. Additionally, two studies were identified as having some concerns regarding bias arising from period and carryover effects.

The certainty of the evidence was evaluated using GRADEpro GDT [34], as detailed in Supplementary Table S2. Certainty for fatigue and vigor outcomes was assessed as low, while certainty for mood state was deemed moderate.

### Effects of β-glucans

Table 2 summarizes the effects of β-glucans on fatigue, vigor, and mood state. Among the ten studies evaluating β-glucans’ effects on fatigue, four demonstrated significantly lower fatigue scores in the β-glucans group compared to the placebo group [8, 9, 25, 30]. These studies targeted populations with mental health issues or regular exercisers, utilized non-grain derived euglena or yeast as sources of β-glucans, administered doses ranging from 250 mg to 500 mg, and had intake durations of four weeks or longer. Among the nine studies evaluating the effects of β-glucans on vigor, three demonstrated significantly higher vigor scores in the β-glucan group compared to the placebo group [8, 29, 30]. These studies targeted populations with mental health issues or regular exercisers, utilized non-grain derived yeast as the source of β-glucans, administered doses ranging from 250 mg to 500 mg, and had intake durations of four weeks or longer. Of the ten studies evaluating the effects of β-glucans on mood state, seven demonstrated significantly higher mood state scores in the β-glucan group compared to the placebo group [8, 22, 26, 28, 29, 35, 36].

**Table 2:** Effects on fatigue, vigor, and mood state.

Study	Outcome
Wu [22]	*Mood state*: The “mental health” score showed no significant difference between the two groups at baseline. The “mental health” score improvement during the intervention period showed limited improvement in the placebo group, whereas a large increase in the β-glucan group was observed. The “mental health” score after 56, 70, and 84 days of intervention showed significantly higher values in the β-glucan group compared to the placebo group (*p* < 0.01).
Wolever [10]	*Fatigue*:
	• The prevalence of fatigue showed no significant difference between the two groups at baseline. The prevalence of fatigue after four weeks of intervention showed no significant difference between the two groups. The prevalence of fatigue after four weeks of intervention showed a significantly lower prevalence in the β-glucan group compared to the baseline (*p* < 0.05).
	• The severity of fatigue after the intervention showed no significant difference between the two groups. The severity of fatigue after four weeks of intervention showed a significant reduction in the β-glucan group compared to the pre-intervention (*p* < 0.05).
Nakashima [23]	*Vigor*: The “vitality” score showed no significant difference between the two groups at baseline. The “vitality” score after 12 weeks of intervention showed a higher value in the β-glucan group (Euglena gracilis powder: 3000 mg) compared to the placebo group.
Kawano [9]	*Fatigue*:
	• The “Overall Fatigue” score showed no significant difference between the two groups at baseline. The change in the “Overall Fatigue” score before and after the intervention showed a lower value in the β-glucan group compared to the placebo group. The “Overall Fatigue” score after four weeks of intervention showed a significantly lower value in the β-glucan group compared to baseline (*p* < 0.01).
	• The change in the “Physical Fatigue” score before and after the intervention showed a significantly lower value in the β-glucan group compared to the placebo group (*p* < 0.05). The “Physical Fatigue” score after four weeks of intervention showed a significantly lower value in the β-glucan group compared to baseline (*p* < 0.01).
	• The change in the “Mental Fatigue” score before and after the intervention showed a significantly lower value in the β-glucan group compared to the placebo group (*p* < 0.05).
Zabriskie [24]	*Fatigue*: The “Fatigue” score showed no significant interaction effect (group × time). The “Fatigue” score after 72 h of exercise showed a significantly lower value in both the β-glucan and placebo groups compared to the score after 0 h of exercise, respectively (*p* < 0.05). The “Fatigue” score after 2 h of exercise showed a significantly lower value in the β-glucan group compared to the score after 0 h of exercise (*p* < 0.05).
	*Vigor*: The “Vigor” score showed a significant interaction effect (group × time) (*p* = 0.04). The “Vigor” score after 72 h of exercise showed a significantly lower value in the placebo group compared to the pre-exercise score (*p* < 0.05).
	Mood state: The “Total Mood Disturbance” score showed a significant interaction effect (group × time) (*p* = 0.007). The “Total Mood Disturbance” score after 72 h of exercise showed significant improvement in the placebo group compared to the score before and 0 h after exercise (*p* < 0.05). The “Total Mood Disturbance” score after 2 h of exercise showed significant improvement in the β-glucan group compared to the score after 0 h of exercise (*p* < 0.05).
Aldwinckle [35]	*Mood state*: The “well-being” score after four weeks of intervention showed improvement in the β-glucan group compared to the placebo group, and the degree of improvement in the β-glucan group showed significant improvement in subjects with lower initial well-being (*p* = 0.0004).
Evans [25]	*Fatigue*:
	• The number of days of fatigue felt from day 1 to day 30 showed a significantly lower value in the β-glucan group compared to the placebo group (*p* = 0.019). The number of days of fatigue felt from day 1 to day 90 showed a lower value in the β-glucan group compared to the placebo group.
	• The severity of fatigue from day 1 to day 30 showed a significantly lower value in the β-glucan group compared to the placebo group (*p* = 0.012). The severity of fatigue from day 1 to day 90 showed a lower value in the β-glucan group compared to the placebo group.
Ishibashi [36]	*Vigor*: The “energy/vitality” score after four weeks of intervention showed a higher value in the β-glucan group compared to the pre-intervention. The placebo group showed a lower value compared to the pre-intervention.
	*Mood state*:
	• The “mental health” score after four weeks of intervention showed a significantly higher value in the β-glucan group compared to the pre-intervention (*p* < 0.05). The placebo group showed a lower value compared to the pre-intervention.
	• The “Mental Component Summary” score after four weeks of intervention showed a significantly higher value in the β-glucan group compared to the placebo group (*p* < 0.05).
Dharsono [26]	*Mood state*:
	• The “psychological sum score” score showed no significant difference between the two groups at baseline. The “psychological sum score” score after 16 weeks of intervention showed a significantly lower value in the placebo group compared to baseline (*p* = 0.0011).
	• The change in “psychological sum score” before and after the intervention showed a significantly lower value in the placebo group compared to the β-glucan group (*p* = 0.038).
Ojiri [47]	*Fatigue*: The “Fatigue” score after ten days of intervention showed a significantly lower value in the β-glucan group compared to baseline (*p* < 0.05). The placebo group showed a higher value compared to baseline.
	*Vigor*: The “Vigor” score after ten days of intervention showed a higher value in the β-glucan group compared to the baseline. The placebo group showed a lower value compared to baseline.
McFarlin [27]	*Mood state*: The “Global Mental Health” score showed no significant difference between the two groups.
Talbott [28]	*Fatigue*: The “Fatigue” score after four weeks of intervention showed no significant difference between the two groups. The “Fatigue” score after four weeks of intervention showed a significantly lower value in the β-glucan group compared to the pre-intervention (*p* < 0.05).
	*Vigor*: The “Vigor” score after four weeks of intervention showed no significant difference between the two groups. The “Vigor” score after four weeks of intervention showed a significantly higher value in the β-glucan group compared to the pre-intervention (*p* < 0.05).
	*Mood state*: The “Global Mood States” score after four weeks of intervention showed significant improvement in the β-glucan group compared to the pre-intervention(*p* < 0.05).
Talbott [29]	*Fatigue*: The “Fatigue” score after 12 weeks of intervention showed a lower value in the β-glucan group compared to the placebo group.
	*Vigor*: The “Vigor” score after 12 weeks of intervention showed a significantly higher value in the β-glucan group compared to the placebo group (*p* < 0.01).
	*Mood state*: The “Global Mood States” score after 12 weeks of intervention showed significant improvement in the β-glucan group compared to the placebo group (*p* < 0.05).
Talbott [30]	*Fatigue*:
	• The “Fatigue” score after 2 and 4 weeks of intervention showed a significantly lower value in the β-glucan group (yeast β-glucan: 500 mg) compared to the placebo group (*p* < 0.05).
	• The “Fatigue” score after four weeks of intervention showed a significantly lower value in the β-glucan group (yeast β-glucan: 250 mg) compared to the placebo group (*p* < 0.05).
	*Vigor*: The “Vigor” score after 2 and 4 weeks of intervention showed a significantly higher value in the β-glucan group (yeast β-glucan: 500 mg) compared to the placebo group (*p* < 0.05).
Talbott [8]	*Fatigue*:
	• The “Fatigue” score after 2 and 4 weeks of intervention showed a significantly lower value in the β-glucan group (yeast β-glucan: 500 mg) compared to the placebo group (*p* < 0.05).
	• The “Fatigue” score after four weeks of intervention showed a significantly lower value in the β-glucan group (yeast β-glucan: 250 mg) compared to the placebo group (*p* < 0.05).
	*Vigor*: The “Vigor” score after 2 and 4 weeks of intervention showed a significantly higher value in the β-glucan group (yeast β-glucan: 500 mg) compared to the placebo group (*p* < 0.05).
	*Mood state*:
	• The “Global Mood State” score after 2 and 4 weeks of intervention showed significant improvement in the β-glucan group (yeast β-glucan: 500 mg) compared to the placebo group (*p* < 0.05).
	• The “Global Mood State” score after four weeks of intervention showed significant improvement in the β-glucan group (yeast β-glucan: 250 mg) compared to the placebo group (*p* < 0.05).
Feldman [31]	*Fatigue*: The number of days of fatigue felt during the intervention period showed no significant difference between the two groups.
	*Vigor*: The SF-36 score showed no significant difference between the two groups.
	*Mood state*: The SF-36 score showed no significant difference between the two groups.

### Meta-analysis

**Fig. 3:**
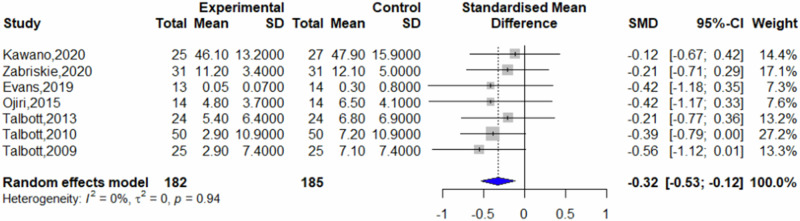
Forest plot on fatigue.

**Fig. 4:**
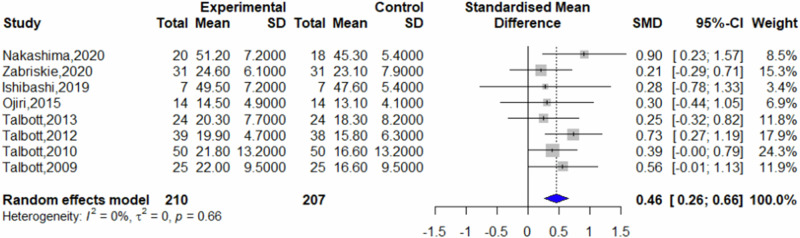
Forest plot on vigor.

**Fig. 5:**
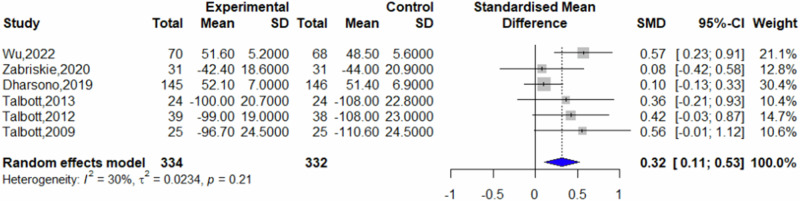
Forest plot on mood state.

Of the included studies, seven reported post-intervention fatigue scores, eight reported post-intervention vigor scores, and six reported post-intervention mood state scores. Other studies did not present data suitable for inclusion in the analysis. Comparative analysis revealed that β-glucans significantly reduced feelings of fatigue compared to placebo (SMD = −0.32, 95% CI = −0.53 to −0.12, *p* = 0.0021, *I*^2^ = 0%) (Fig. 3). Furthermore, β-glucans significantly increased vigor compared to placebo (SMD = 0.46, 95% CI = 0.26–0.66; *p* < 0.0001, *I*^2^ = 0%) (Fig. 4), and improved mood state significantly (SMD = 0.32, 95% CI = 0.11 to 0.53; *p* = 0.0026, *I*^2^ = 30%) (Fig. 5).

Analysis of funnel plots and Begg and Egger tests indicated no evidence of publication bias (*p* > 0.1) (Supplementary Fig. S1).

### Subgroup analysis

Subgroup analyses were conducted based on outcome evaluation methods for fatigue, vigor, and mood state. Meta-analysis was performed only on studies where the evaluation method was POMS, as the number of studies in other subgroups was two or fewer.

Compared to the placebo group, β-glucans significantly reduced feelings of fatigue (SMD = −0.35, 95% CI = −0.58 to −0.12, *p* = 0.0031, *I*^2^ = 0%) (Supplementary Fig. S2). Furthermore, β-glucans significantly increased vigor compared to placebo (SMD = 0.43, 95% CI = 0.22–0.63; *p* < 0.0001, *I*^2^ = 0%) (Supplementary Fig. S3), and significantly improved mood state compared to placebo (SMD = 0.35, 95% CI = 0.09–0.60; *p* = 0.0082, *I*^2^ = 0%) (Supplementary Fig. S4).

## Discussion

This study delved into the effects of β-glucans on fatigue across 16 studies involving healthy subjects. A meta-analysis based on 12 of these studies revealed significant reductions in feelings of fatigue (SMD = −0.32, 95% CI = −0.53 to −0.12; *p* = 0.0021), significant increases in vigor (SMD = 0.46, 95% CI = 0.26–0.66; *p* < 0.0001), and significant improvements in mood state (SMD = 0.32, 95% CI = 0.11–0.53; *p* = 0.0026). These findings suggest that β-glucans are effective in alleviating fatigue among healthy individuals.

To effectively reduce feelings of fatigue, it may be necessary to take β-glucans for at least four weeks. Additionally, there may be potential benefits for individuals under physical or psychological stress, such as those dealing with mental health issues or engaging in regular exercise.

Strenuous exercise has been associated with adverse effects on various immune parameters, potentially increasing susceptibility to infections and other ailments [37]. Similarly, excessive stress can impact the autonomic nervous and immune systems, leading to poor sleep quality and reduced work efficiency [38, 39]. β-glucans derived from yeast have been shown to activate immune cells and promote the production of cytokines and antibodies [40, 41], which may contribute to their fatigue-reducing effects. β-glucans are recognized via receptors such as Dectin-1 and CR3, which activate innate and adaptive immunity [40]. Additionally, studies have indicated associations between fatigue and immune parameters such as sIgA concentrations, further supporting the immunostimulatory role of β-glucans [42, 43]. Moreover, β-glucans sourced from euglena have demonstrated significant increases in sIgA concentration secretion rates in healthy subjects [36], reinforcing their potential in mitigating fatigue through immune modulation.

Interestingly, while studies involving individuals with moderate psychological stress showed non-significant group differences in fatigue scores [29], those with moderate to severe psychological stress exhibited significant group differences [30]. This suggests that as psychological stress levels intensify, the impact of β-glucans on fatigue may become more pronounced.

When synthesizing results, it’s essential to consider variations in outcome assessment methods. In this study’s meta-analysis, the same concepts (such as fatigue and vigor) were evaluated using different methods. It is recommended that when synthesizing results, differing evaluation methods for the same concepts should not be relied upon as the sole approach [44]. Therefore, subgroup analyses based on differences in outcome evaluation methods were conducted. Meta-analysis of studies utilizing the Profile of Mood States (POMS) scale demonstrated that β-glucans significantly reduced feelings of fatigue, increased vigor, and improved mood state compared to placebo. These findings suggest the effectiveness of β-glucans in alleviating feelings of fatigue among healthy individuals.

Furthermore, oxidative stress and antioxidant capacity measurements offer valuable insights into fatigue status [45]. In a study evaluating oxidative stress, markers of antioxidant capacity (BAP) and BAP/d-ROMs ratio were significantly higher in the β-glucan group compared to placebo at four weeks post-intervention. Moreover, the change in BAP levels before and after the intervention was also significantly higher in the β-glucan group [9]. Additionally, Salecan, a β-glucan derived from soil bacteria, has been shown to modulate antioxidant defense mechanisms in animal models [46]. These findings suggest that the antioxidant properties of β-glucans may contribute to their fatigue-reducing effects.

Several limitations warrant consideration in interpreting the findings of this study. Firstly, heterogeneity among studies, stemming from differences in target populations, sources of β-glucans, intake regimens, durations, and outcome measures, may have influenced result accuracy. Future research, including meta-regression analysis, should explore optimal β-glucan intake strategies. Secondly, concerns regarding bias risk and publication bias exist. Only two studies were classified as “low risk” for overall bias among the included studies. Although Begg and Egger tests did not reveal significant differences, the limited number of studies precludes definitive conclusions regarding publication bias. Lastly, the number of studies and sample sizes in this analysis may be insufficient. Further clinical trials with larger sample sizes are warranted to validate the findings and provide robust evidence for the efficacy of β-glucans in managing fatigue.

## Conclusion

In conclusion, the findings from this systematic review and meta-analysis suggest that β-glucans hold promise as a potential intervention for reducing feelings of fatigue among healthy individuals. However, given the limitations identified, including heterogeneity among studies and concerns regarding bias risk and publication bias, further clinical trials are necessary to confirm and validate these findings. Additionally, considering the limited number of studies included in this analysis, larger-scale trials are warranted to provide more robust evidence regarding the efficacy of β-glucans in fatigue management.

## Supplementary information

Supplementary Material_Revised_Clean

## Data Availability

The data utilized for all analyzes conducted in this manuscript are available from the figures and tables presented herein. Furthermore, the R package used for statistical analyses is accessible online, ensuring transparency and reproducibility of the findings.
